# Barriers to access and utilization of emergency obstetric care at health facilities in sub-Saharan Africa—a systematic review protocol

**DOI:** 10.1186/s13643-018-0720-y

**Published:** 2018-04-16

**Authors:** Ayele Geleto, Catherine Chojenta, Abdulbasit Mussa, Deborah Loxton

**Affiliations:** 1 School of Public Health, College of Health and Medical Sciences, Haramaya University, Harar, Ethiopia; 2 Research Centre for Generational Health and Ageing, School of Medicine and Public Health, Faculty of Health and Medicine, The University of Newcastle, Newcastle, Australia; 3 School of Nursing and Midwifery, College of Health and Medical Science, Haramaya University, Harar, Ethiopia

**Keywords:** Emergency obstetric care, Barrier, Access, Utilization, Sub-Saharan Africa

## Abstract

**Background:**

Nearly 15% of all pregnancies end in fatal perinatal obstetric complications including bleeding, infections, hypertension, obstructed labor, and complications of abortion. Between 1990 and 2015, an estimated 10.7 million women died due to obstetric complications. Almost all of these deaths (99%) happened in developing countries, and 66% of maternal deaths were attributed to sub-Saharan Africa. The majority of cases of maternal mortalities can be prevented through provision of evidence-based potentially life-saving signal functions of emergency obstetric care. However, different factors can hinder women’s ability to access and use emergency obstetric services in sub-Saharan Africa. Therefore, the aim of this review is to synthesize current evidence on barriers to accessing and utilizing emergency obstetric care in sub-Saharan African. Decision-makers and policy formulators will use evidence generated from this review in improving maternal healthcare particularly the emergency obstetric care.

**Methods:**

Electronic databases including MEDLINE, CINAHL, Embase, and Maternity and Infant Care will be searched for studies using predefined search terms. Articles published in English language between 2010 and 2017 with quantitative and qualitative design will be included. The identified papers will be assessed for meeting eligibility criteria. First, the articles will be screened by examining their titles and abstracts. Then, two reviewers will review the full text of the selected articles independently. Two reviewers using a standard data extraction format will undertake data extraction from the retained studies. The quality of the included papers will be assessed using the mixed methods appraisal tool. Results from the eligible studies will be qualitatively synthesized using the narrative synthesis approach and reported using the three delays model. The Preferred Reporting Items for Systematic Reviews and Meta-Analyses checklist will be employed to present the findings.

**Discussion:**

This systematic review will present a detailed synthesis of the evidence for barriers to access and utilization of emergency obstetric care in sub-Saharan Africa over the last 7 years. This systematic review is expected to provide clear information that can help in designing maternal health policy and interventions particularly in emergency obstetric care in sub-Saharan Africa where maternal mortality remains high.

**Systematic review registration:**

PROSPERO CRD42017074102.

**Electronic supplementary material:**

The online version of this article (10.1186/s13643-018-0720-y) contains supplementary material, which is available to authorized users.

## Background

Globally, nearly 15% of all pregnant women face at least one lethal perinatal obstetric complication [1]. Many mothers with obstetric complications may die if left untreated. According to the World Health Organization’s (WHO) 2015 report on maternal mortality trends, an estimated 10.7 million women died from 1990 to 2015 due to obstetric causes. The report showed that globally in 2015 alone an estimated 303,000 women died during pregnancy and childbirth [2]. Similarly, the 2014 WHO maternal mortality fact sheet reported that an estimated 800 women die every day from pregnancy related complications. Almost all of these deaths (99% of global maternal mortalities) occurred in developing countries, and 66% of these deaths were attributed to sub-Saharan Africa countries [3].

Preventable complications arising during pregnancy and childbirth, including bleeding, infections, hypertension, obstructed labor and unsafe abortion, can end in maternal death [4–8]. Provision of evidence-based quality Emergency Obstetric Care (EmOC) helps to prevent maternal deaths [9–12]. There are two complementary types of EmOC: Basic Emergency Obstetric Care (BEmOC) and Comprehensive Emergency Obstetric Care (CEmOC) [13]. A BEmOC facility is expected to provide six crucial obstetric services known as “signal functions” which includes administration of parenteral antibiotics, parenteral anticonvulsants, parenteral uterotonics, removal of retained products, manual removal of placenta, and assisted vaginal delivery (AVD). A CEmOC facility provides cesarean section and blood transfusion in addition to the signal functions of BEmOC [14].

Implementation of emergency obstetric care has resulted in a noticeable global decline in the maternal mortality ratio (MMR) since 1990, though the reduction varies across regions of the world [15]. Over the past 25 years, MMR has fallen by 43% with an average annual decline rate of 3.0% between 2000 and 2015. However, the observed annual decline rate is still far below the global Millennium Development Goals (MDG) target for which the yearly reduction rate would be 5.5% and significant regional variation [2]. Sub-Saharan Africa still sustains a huge MMR. For example, in Ethiopia MMR was 412 per 100,000 live births in 2015 even though the MDG target is to reduce MMR to less the 267 per 100,000 live births by 2015 [16]. The United Nations (UN) has set a new global strategy, the Sustainable Development Goals (SDGs) that is aimed at reducing the global MMR to less than 70 per 100,000 live births and having no country with more than twice the global target by 2030 [17]. This target will not be achieved unless provision of quality EmOC is strengthened, especially in sub-Saharan Africa where MMR is comparatively higher.

Research results from developing countries have indicated poor utilization of EmOC among women who have experienced obstetric complications. For instance, the study of Freedman et al. in 2007 of nine sub-Saharan African countries indicated met need for EmOC was only 28% [18]. A recent study in Tanzania indicated that only 22% of mothers in need of EmOC were able to obtain EmOC services [19]. In Zambia, unmet need for EmOC was reported being as high as 73% [20], while met need of EmOC for Malawian women was 20.7% [21]. In Ethiopia, even though the target of met need for EmOC was set to be 75% by 2015 [22], the study by Worku and colleagues has indicated 52.1% met need for EmOC [4] while a former findings of Admasu et al. showed only 6% of Ethiopian women requiring EmOC were treated at health institutes [23].

Several factors are reported to determine access and utilization of EmOC services in sub-Saharan Africa. While EmOC service availability remains a factor [21, 24–26], numerous other barriers to EmOC service utilization have been reported, such as lack of knowledge about pregnancy complications [27–30] and poor awareness of availability of EmOC service [31–34]. The quality of the service is also an important determinant in deciding to use EmOC services. The majority of studies conducted in sub-Saharan Africa noted the poor quality of EmOC as indicated by reported direct obstetric case fatality rates above the UN threshold of 1% [6, 21, 24, 35, 36]. Lack of essential supply and poor providers’ competence [37] and lack of training for healthcare providers [35] that affect quality of EmOC service were also reported.

Limited previous reviews exist on obstetric health services in sub-Saharan Africa, and no review to date have specifically focused on barriers to access and utilization of EmOC services. While some reviews have focused on access barriers to obstetric care [38] these have not specifically been in relation to EmOC. Further, others have focused on facilitators and barriers of facility delivery [39] and other studies have focused on the application of international guidelines for emergency obstetric care [40]. Therefore, this systematic review will identify and present factors affecting access to and utilization of EmOC using the three delay model [41] and Popay et al.’s narrative synthesis guideline [42]. The aim of this review is to synthesize current evidence on barriers to access and utilization of EmOC at health institutions in sub-Saharan Africa. The findings will be used as an input for decision-makers and policy formulators to plan and implement evidence-based maternal health services, particularly the EmOC.

## Methods

### Data sources

A systematic search for appropriate published articles on barriers to access and utilization of emergency obstetric care in sub-Saharan Africa will be conducted. The findings of eligible studies will be systematically presented using a narrative synthesis approach. The study protocol of this review was registered in PROSPERO 2017: ID = CRD42017074102 (available at http://www.crd.york.ac.uk/PROSPERO/display_record.asp?ID=CRD42017074102). Online databases including MEDLINE, CINAHL, Embase, and Maternity and Infant Care will be searched to identify appropriate articles. All identified papers from the selected databases will be comprehensively assessed for meeting eligibility criteria. The Preferred Reporting Items for Systematic Reviews and Meta-Analyses (PRISMA) checklist [43] (Additional file 1) will be employed to present the findings of studies on barriers to utilization of EmOC in sub-Saharan Africa.

### Search strategy

In consultation with the faculty librarian, we will retrieve articles published in the English language between 2010 and 2017 (current) from the online databases. This systematic review will use the PICOS (Population, Intervention, Comparison, Outcomes and Study setting) framework to decide the eligibility of the articles. Participant (*P*) refers to mothers who experienced any one of obstetric complications who do not accessed to EmOC while the Intervention **(***I***)** is emergency obstetric care. Comparison (*C*) is those mothers who experienced any one of obstetric complications and received EmOC, the outcome (*O*) is barriers to access and utilization of EmOC while the study setting (*S*) is sub-Saharan Africa.

We will include papers published from 2010 in order to capture barriers to access and utilization of emergency obstetric care after the release of the updated handbook for monitoring emergency obstetric care [13]. The following combination of search terms and strategy will be used to locate suitable articles from the identified databases:

“Emergency Obstetric Care” OR “Emergency Obstetric and Newborn Care” OR EmOC OR EmONC OR “pregnancy complication*” OR “obstetric complication*” OR “maternal ha?morrhage” OR “pregnancy induced hypertension” Eclampsia OR Pre-eclampsia OR “maternal infection” OR “obstructed labo?r” OR “complication* of abortion” OR “cesarean section” OR “manual vacuum extraction” OR Oxytocin OR “magnesium sulphate” AND (barrier* OR obstacle* OR factor* OR Challenge* OR determinant* OR access* OR utiliz* OR Utilis* OR hinder* OR hindrance* OR impede* OR impediment*).mp. AND “sub-Saharan Africa” OR “Africa South of Sahara” to locate relevant articles for this systematic review. The reference lists of eligible papers will also be searched manually, and if they are found suitable for the review, they will be included. MEDLINE database searching strings and strategy are presented in Additional file 2. The identified articles will be screened guided by the inclusion and exclusion criteria.

### Screening of the articles

Results from the initial searches will be stored in an EndNote library. After removing duplicated articles, the EndNote library will be shared between the two reviewers to independently screen the articles by title and abstract, guided by the eligibility criteria. Those studies which the two reviewers agreed on will be proceed to the full text review. A third reviewer will adjudicate any discrepancies between the two reviewers. Two reviewers will independently review the full text of all eligible papers. In the case where there are differences between the two reviewers, consensus will be sought through discussion on the differences with a third reviewer. Finally, the full texts of all relevant articles found to meet the inclusion criteria will be retained for the final narrative synthesis.

### Eligibility criteria

The following criteria will be used to select articles that will be included in to our systematic review. All peer-reviewed articles published in English, reporting barriers to access and utilization of EmOC from service users’ perspective and challenges to provide emergency obstetric services at health facilities will be included. That is, all articles that reported any factors that delay mothers at home, on the way to the health facility and at the facility in receiving timely services will be included into the review. We will include both quantitative and qualitative studies and they must have been conducted in sub-Saharan Africa countries. The quantitative studies should have been conducted with case-control, cohort, and cross-sectional designs. To be included for the study, the data collection period should have been from January 2010 to August 2017. All studies irrespective of the setting where they were conducted will be included.

### Exclusion criteria

Articles published in languages other than English and data collection period took place before January 2010 will be excluded from our review in order to capture access barriers to EmOC after the release of updated handbook for monitoring EmOC [13]. We will exclude studies that report barriers to utilization of obstetric care that have women with no obstetric complications as the population of interest, due to the fact that women with obstetric complications are more likely to seek special services than women without obstetric complications. Commentaries and anonymous reports will be excluded from this systematic review.

### Quality appraisal

The mixed methods appraisal tool (MMAT) [44] by Pluye and colleagues will be used to assess the quality of identified papers. This quality assessment tool was found to have moderate to perfect interrater reliability score [45] and has been used in different systematic reviews that included studies conducted with quantitative, qualitative and mixed method designs [38, 46]. The MMAT was designed to assess the quality of articles conducted with qualitative, quantitative and mixed methods designs. The tool allows to concurrently appraising the quantitative method of randomized controlled trial, nonrandomized and descriptive studies. According to Pluye et al. (2011), all of these designs have their own criteria and studies are scored by dividing the number of criteria met by total criteria to arrive at a quality percentage. There are four quality assessment criteria for each studies conducted with qualitative and quantitative methods and the quality of each study is determined by dividing the number of criteria met by four. The quality value ranges from 25% (one criteria met) to 100% (all criteria met). For mixed methods studies, the premise is that the overall quality of a combination cannot exceed the quality of its weakest component. Thus, the overall quality score for mixed methods designs is the lowest score of the study components. The quality score is 25% when qualitative = 1 or quantitative = 1 or mixed method = 0; it is 50% when qualitative = 2 or quantitative = 2 or mixed method = 1; it is 75% when qualitative = 3 or quantitative = 3 or mixed method = 2; and it is 100% when qualitative = 4 and quantitative = 4 and mixed method = 3 [47]. Since the aim of this review is to identify barriers to utilization of emergency obstetric care, all identified studies will be included irrespective of their quality scores. However, the quality score of each paper will be reported separately in the results.

### Data extraction

Data will be extracted from the full text of retained articles using an adapted Johanna Briggs Institute (JBI) data abstraction format [48] (Table 1). Study characteristics to be extracted will include name of the first author and publication year, data collection period and the country in which the study was conducted. Specific study details including study design, study population, sample size, sampling procedure, data collection procedure and response rate will then be captured. Factors reported as barriers/challenges to access and utilization of emergency obstetric care will be systematically identified.

**Table 1:** Data extraction format

Study characteristics	
Author and year	
Title of the study	
The journal	
Reviewer	
Objective of the study	
Study participant	
Country of the study	
Study design	
Sample size	
Sampling technique	
Data collection method	
Data collection period	
Data analysis method	
Ethically approved from	
Quality score of the article	
Relevant findings	
Author’s conclusion	
Reviewer’s comments	

### Data synthesis

The information on barriers to access and utilization of emergency obstetric care obtained from the final full text articles will be summarized using narrative synthesis. Since we will not conduct a meta-analysis, the findings of studies conducted with qualitative, quantitative and mixed method designs will be summarize and presented in a narrative style. The guideline of Popay and colleagues [42] used to conduct narrative synthesis in systematic reviews will be employed to conduct the narrative synthesis. This guidance has four elements including: (1) developing a theory, (2) developing a preliminary synthesis, (3) exploring relationships within and between studies, and (4) assessing the robustness of the synthesis.

The basic theory that will be applied in this review is identification of barriers to the access and utilization of emergency obstetric care. The identified barriers will be retrieved using the three delays model of Thaddeus and colleague [41] that is based on three broad categories (first delay, second delay, and third delay). This theoretical framework can capture any factors that causes delay in seeking healthcare and was employed in different scientific papers [33, 49]. As part of developing a preliminary synthesis, summary tables will be generated from crude data on barriers to the access and utilization of emergency obstetric care. To explore relationships within and between studies, findings will be grouped into themes qualitative case descriptions will be performed. Finally, to assess the robustness of the synthesis, validity assessment and quality appraisal of the papers will be critically employed. At all stages of the review process, including searching articles, extraction and the narrative synthesis, appropriate standards will be strictly applied. Article screening and the selection process of the reviewed studies will be illustrated using a PRISMA flow diagram [43] (Additional file 3).

## Discussion

This systematic review will provide a detailed summary of the evidence for the barriers to access and utilization of emergency obstetric care in sub-Saharan African countries over a 7-year period. Several studies findings have indicated that most of the sub-Saharan Africa region did not achieve the MDGs of 75% reduction in maternal mortality by the end of 2015. This might be attributed to the poor utilization of emergency obstetric care as reported from different developing countries [6, 12, 21, 50, 51]. Factors including unavailability of EmOC signal functions at health facilities [4, 23, 25, 26], lack of awareness about pregnancy complications [27–30], poor educational status, and lack of media exposure [52] are reported to affect access to and utilization of EmOC services among women in need of it. In developing countries, different socio-cultural and economic factors as well as poor health infrastructure are contributing to the poor access and utilization of EmOC services. Therefore, this review will systematically identify, synthesize and present the most common barriers and challenges that hinder women from accessing and using EmOC. The review will provide clear information required for designing maternal health interventions particularly EmOC in sub-Saharan Africa where maternal mortality is significantly higher.

## Additional files

Additional file 1: PRISMA 2009 Checklist. PRISMA checklist for reporting of systematic review. (DOC 62 kb)

Additional file 2: MEDLINE search strategy. Sample search strategy (for MEDLINE database). (DOCX 13 kb)

Additional file 3: PRISMS 2009 Flow Diagram. Flow chart indicating screening of the articles. (DOCX 26 kb)

## Abbreviations

AVD: Assisted vaginal delivery
BEmOC: Basic Emergency Obstetric Care
CEmOC: Comprehensive Emergency Obstetric Care
CINAHL: Cumulative Index to Nursing and Allied Health Literature
EmOC: Emergency Obstetric Care
MDGs: Millennium Development Goals
MMAT: Mixed method appraisal tool
MMR: Maternal mortality rate
PRISMA: Preferred Reporting Items for Systematic Reviews and Meta-Analyses
SDGs: Sustainable Development Goals
UNs: United Nations
WHO: World Health Organization
