# Supplementary material for: Barriers to access and utilization of emergency obstetric care at health facilities in sub-Saharan Africa—a systematic review protocol
Source: Syst Rev. 2018 Apr 16;7:60. doi: 10.1186/s13643-018-0720-y (PMC5902829; doi:10.1186/s13643-018-0720-y)
Supplement: Supplementary file 3 — PRISMS 2009 Flow Diagram. Flow chart indicating screening of the articles. (DOCX 26 kb) [file 13643_2018_720_MOESM3_ESM.docx]

## Included

## Eligibility

## Screening

## Identification

Records identified through snowball search (n =)

Records identified through database search (n =)

All records retrieved (n =)

Duplicate removed (n =)

Articles screened by title and abstract (n =)

Records removed based on title and abstract (n =)

Full text articles assessed for eligibility (n =)

Full text articles included in qualitative synthesis (n =)

Full-text articles excluded (n =)

- Reason one (n = )
- Reason two (n = )
- Reason three (n= )
- Reason four (n = )
- Reason five (n = )
